# Implementation of a telephone-based secondary preventive intervention after acute coronary syndrome (ACS): participation rate, reasons for non-participation and 1-year survival

**DOI:** 10.1186/s13063-016-1203-x

**Published:** 2016-02-15

**Authors:** Daniel Huber, Robin Henriksson, Stina Jakobsson, Nikolai Stenfors, Thomas Mooe

**Affiliations:** Department of Public Health and Clinical Medicine, Centre of Medicine Östersund, Umeå University, Umeå, Sweden

**Keywords:** Secondary prevention, Acute coronary syndrome, Myocardial infarction, Cardiovascular disease, Implementation, Telemedicine, Telephone, Nurse-based, Mortality, Randomized controlled trial

## Abstract

**Background:**

Acute coronary syndrome (ACS) is a major cause of death from a non-communicable disease. Secondary prevention is effective for reducing morbidity and mortality, but evidence-based targets are seldom reached and new interventional methods are needed. The present study is a feasibility study of a telephone-based secondary preventive programme in an unselected ACS cohort.

**Methods:**

The NAILED (Nurse-based Age-independent Intervention to Limit Evolution of Disease) ACS trial is a prospective randomized controlled trial. All eligible patients admitted for ACS were randomized to usual follow-up by a general practitioner or telephone follow-up by study nurses. The intervention was made by continuous telephone contact, with counseling on healthy living and titration of medicines to reach target values for blood pressure and blood lipids. Exclusion criteria were limited to physical inability to follow the study design or participation in another study.

**Results:**

A total of 907 patients were assessed for inclusion. Of these, 661 (72.9 %) were included and randomized, 100 (11 %) declined participation, and 146 (16.1 %) were excluded. The main reasons for exclusion were participation in another trial, dementia, and advanced disease. “Excluded” and “declining” patients were significantly older with more co-morbidity, decreased functional status, and had more seldom received education above compulsory school level than “included” patients. Non-participants had a higher 1-year mortality than participants.

**Conclusions:**

Nurse-led telephone-based follow-up after ACS can be applied to a large proportion in an unselected clinical setting. Reasons for non-participation, which were associated with increased mortality, include older age, multiple co-morbidities, decreased functional status and low level of education.

**Trial registration:**

International Standard Randomized Controlled Trial Number (ISRCTN): ISRCTN96595458 (archived by WebCite at http://www.webcitation.org/6RlyhYTYK). Application date: 10 July 2011.

## Background

Survival has improved remarkably for acute cardiovascular disease over the last few decades due to improved treatment, evidence-based guidelines and effective medications for reducing secondary morbidity. However, cardiovascular disease is still the major cause of death from a non-communicable disease, accounting for 17.3 million deaths annually worldwide, four million in Europe alone. The total cost for society is estimated to be US$863 billion worldwide and US$190 billion in Europe annually [1].

The risk of death, recurrent acute coronary syndrome (ACS) or stroke/transitory ischemic attack (i.e., cerebrovascular lesion, CVL) is markedly increased after an initial cardiac ischemic event [2]. The most important aspect for reducing mortality after ACS is secondary preventive measures [3, 4]. To address this, evidence-based guidelines on secondary intervention after cardiovascular disease have been issued both nationally and internationally [5]. Medical treatment alone has been shown to reduce the risk of re-infarction and death by 20–30 % [6]. However, multiple studies have shown that compliance to guidelines is surprisingly low [7, 8] but increasing slightly [4]. In Sweden in 2013, only 12 % of ACS patients in the SWEDEHEART registry reached targets for the four most important preventable risk factors at follow-up (i.e., smoking, blood pressure, low-density lipoprotein (LDL) and physical exercise) [9]. In the EUROASPIRE IV survey, only one fifth of those on lipid-lowering medication reached the target for LDL cholesterol (<1.8 mmol/L), and less than one third achieve targets for blood pressure, despite a high prevalence of modern medication use [10].

Thus, a more effective method of achieving secondary prevention targets is needed [11]. One method, which has proven to be cost-effective, is follow-up and medical titration via telemedical methods [12–14]. The NAILED (Nurse-based Age-independent Intervention to Limit Evolution of Disease) study is an on-going randomized controlled study on secondary preventive measures after ACS and CVL. The aim of the NAILED study was to evaluate whether nurse-based follow-up via telephone is a more effective method of reaching set target values for blood lipids and blood pressure than ordinary care by a general practitioner (GP) [15]. The aim of this present study was to examine the feasibility of the NAILED protocol in ACS patients on a population basis. We examined the rate of participation, rate and reasons for non-participation, differences between the groups and the 1-year mortality rate.

## Methods

The overall NAILED study was designed as a non-blinded prospective randomized controlled trial. Participants in the present study comprised all who were considered for inclusion in the NAILED ACS risk factor trial between 1 January 2010 and 31 January 2013. The exclusion criteria were limited to an inability to adhere to a telephone intervention and participation in another trial. The trial was conducted at Östersund County hospital, Jämtland, Sweden, the only hospital in the county. The hospital has a rural catchment area of approximately 126,000 inhabitants, and is where all patients with suspected ACS in the county are sent. The ACS diagnosis was defined as myocardial infarction type 1 [16], comprising ST-elevation myocardial infarction (STEMI) and non-ST-elevation infarction (NSTEMI) or unstable angina (UA) based on symptoms of myocardial ischemia together with electrocardiographic changes (ST depression or T wave changes) indicative of myocardial ischemia.

During in-hospital care, all patients diagnosed with ACS were evaluated based on baseline data in interview and medical records (i.e., clinical status, risk factors and co-morbidities) by specially trained study nurses. Patients eligible for inclusion at discharge were randomized to follow-up as usual by a GP (control) or by a study nurse (case). An extensive description of the study design for the randomized trial is available in the study protocol [15]. Briefly, all cases were contacted by telephone 1 month after discharge and then annually for 3 years with prior measurements of standardized blood pressure and appropriate blood specimens. The cases were counseled on compliance, smoking cessation and exercise, and their medication titrated to reach set targets for blood pressure and lipids. A study physician made decisions about interventions. Follow-up occurred a month after every intervention, and a new titration was made if necessary. Unscheduled appointments could be made at the patient’s request. Controls had their standardized blood pressure and the same blood samples taken at the same interval, and the results were reported to their GP and the study nurses. The aim of the overall study is to evaluate the hypothesis that a nurse-conducted telephone-based approach to secondary prevention will lead to a significantly larger proportion of patients reaching set targets for blood pressure and LDL cholesterol compared to usual practice.

The following reasons for exclusion were documented: participation in another on-going medical trial or not being able to participate according to the study design (i.e., not being physically or cognitively able to handle a telephone or unable to commute to have blood samples drawn). A 1-year follow-up of mortality was performed in all groups for assessment. Causes of mortality were classified based on the national Swedish National Cause of Death Register or, if insufficient, medical records. Kidney function was measured as estimated glomerular filtration rate (eGFR) based on the creatinine value at admission using the CKD-EPI formula. Every patient’s functional status was assessed according to the modified Rankin scale (mRS) by the study nurses.

Statistical analyses were performed with IBM SPSS statistics software v 22.

All patients, or their next of kin, gave verbal informed consent to the collection of baseline data and the study was approved by the Regional Ethical Review Board, Umeå University, Umeå, Sweden (16/12/2009, ref: Dnr 09-142 M; 10/06/2013, ref: Dnr 2013-204-32 M).

### Statistical analysis

Patients were subdivided into the following categories: “included” (eligible for inclusion and willing to participate), “declined” (eligible for inclusion but not willing to participate) and “excluded” (not eligible for inclusion). We compared basic characteristics and variables of interest between the three groups with two-sided chi^2^ tests, Fischer’s exact test or independent samples *t* test as appropriate. A *p* value <0.05 was considered significant.

To identify independent predictors of the decision to not participate, we set up a multivariate logistic regression model of variables with an alpha level <0.1 in the univariate analyses between the “included” and “declined” groups. We then performed manual stepwise exclusion based on the level of significance. To evaluate independent baseline characteristics important for exclusion, we set up a second multivariate model between included and excluded patients in the same manner. Sex and age were included in both regression models regardless of statistical significance. The results are presented as odds ratios (ORs) with 95 % confidence intervals (CIs). We categorized continuous variables in the multivariate models.

To assess 1-year cumulative survival we made Kaplan-Meier estimations with group comparisons using the log rank test. To calculate ORs for mortality, we used univariate logistic regression.

## Results

**Fig. 1:**
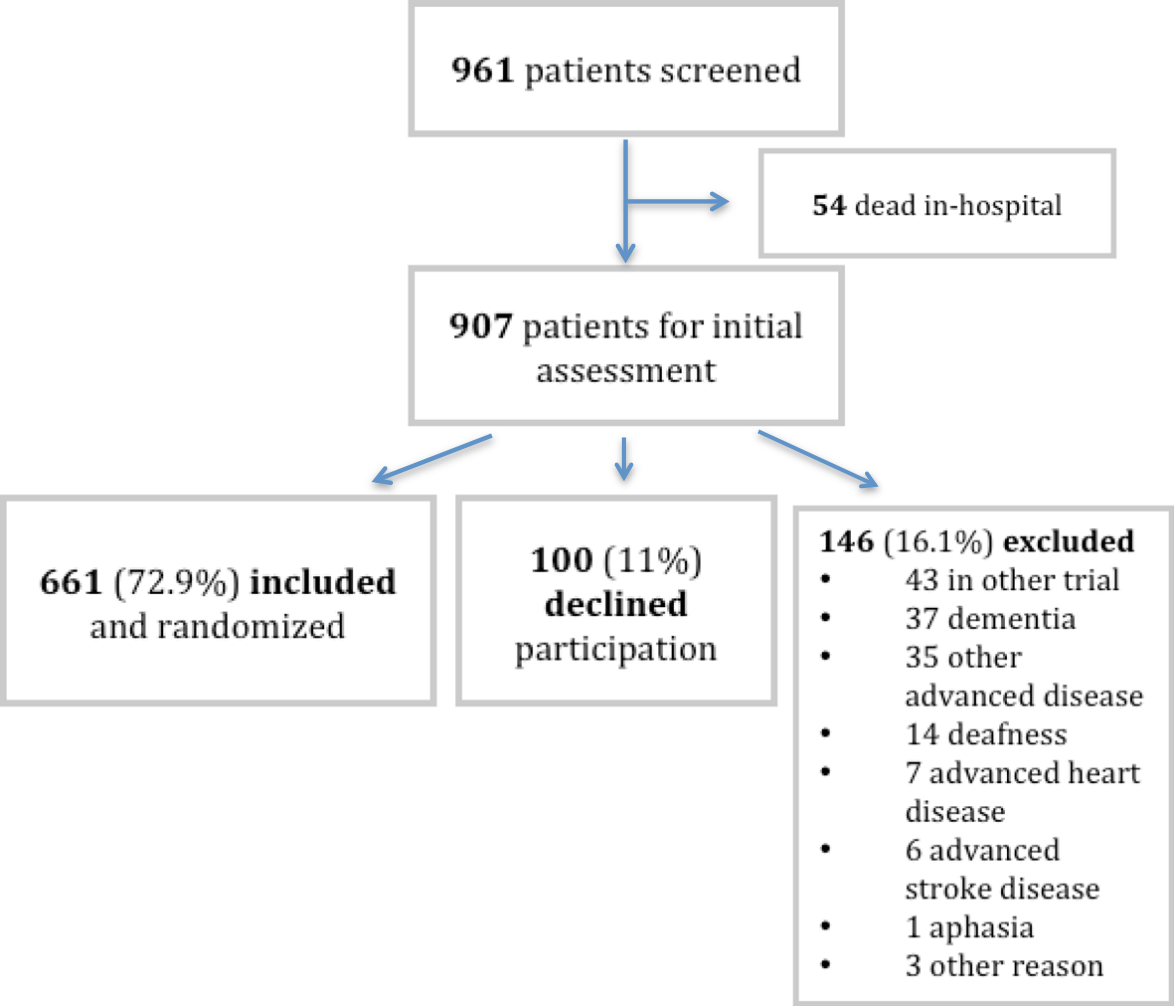
Study flow chart

During the inclusion period, 961 patients were hospitalized with ACS and primarily assessed. Fifty-four patients died during hospitalization. Of the remaining 907 patients, 249 (27.5 %) were diagnosed with STEMI, 589 (64.9 %) with NSTEMI, and 69 (7.6 %) with unstable angina (UA). After assessment, 661 (73 %) were randomized into the study, 146 (16 %) were excluded, and 100 (11 %) declined participation (Fig. 1). The most common cause for exclusion was participation in another trial (29 %, 43/146), followed by dementia (25 %, 37/146) and advanced disease other than cardiac disease or stroke (24 %, 35/146). Taking into account the eligible patients excluded because of participation in another trial, the “excluded” group would decrease to 103 patients (11 %).

Baseline patient characteristics are presented in Table 1. In a univariate comparison between the “included” and “excluded” subgroups, we found that the “excluded” group consisted of significantly older patients and a larger proportion with a mRS score >3. The “excluded” group had a larger proportion of women and a lower proportion of patients with an education above compulsory school level. Excluded patients had a lower body mass index (BMI), decreased kidney function, and a larger proportion with previous ischemic heart disease (IHD), congestive heart failure (CHF), stroke and peripheral arterial disease. The “included” group also had fewer patients with atrial fibrillation, prior hypertension or diabetes. No variables were missing more than 3 % data except “known hyperlipidemia” (missing 25.6 % of data) which, therefore, was excluded from all further analyses.

**Table 1:** Patient characteristics

	Included		Excluded		Declined		*p*	*p*	*p*
	*N*	% or SD	*n*	% or SD	*n*	% or SD	i/e	i/d	d/e
Subjects	661	72.9	146	16.1	100	11			
Gender (male)	453	68.5	80	54.8	47	53	0.002	<0.001	0.230
Mean age (years ± SD)	69.2 ± 11.9		76.3 ± 12.1		77.3 ± 11.7		<0.001	<0.001	0.499
Basic characteristics									
mRS >3	7	1.1	33	22.6	8	8.0	<0.001	<0.001	0.003
eGFR	78.4 ± 21.3		68.9 ± 22.9		70.4 ± 21.5		<0.001	0.001	0.597
eGFR <60	128	19.4	52	35.6	29	29	<0.001	0.026	0.278
eGFR 60–90	308	46.6	66	45.2	50	50	0.76	0.525	0.459
eGFR >90	225	34.0	28	19.2	21	21	<0.001	0.009	0.725
BMI (mean ± SD)	27.3 ± 4.5		25.8 ± 5.2		26.4 ± 5		0.001	0.077	0.375
BMI <18.5	5 ± 0.8		14 ± 10.1		3 ± 3.1		<0.001	0.070	0.044
BMI 18.5–25	206 ± 31.2		49 ± 35.3		34 ± 35.1		0.347	0.442	0.975
BMI 25–30	295 ± 44.6		53 ± 38.1		39 ± 40.2		0.160	0.413	0.748
BMI >30	155 ± 23.4		23 ± 16.5		21 ± 21.6		0.075	0.695	0.322
Only basic education	345	52.2	99	67.8	77	77.0	<0.001	<0.001	0.231
Heredity (first line)	178	27.3	28	21.5	20	21.5	0.173	0.237	0.995
Previous morbidities									
Previous IHD	156	23.6	51	34.9	34	34.0	0.005	0.025	0.880
Current NSTEMI	420	63.5	100	68.5	69	69.0	0.258	0.288	0.933
Current STEMI	182	27.5	42	28.8	25	25.0	0.763	0.596	0.514
Previous stroke	48	7.3	26	17.8	15	15.0	<0.001	0.009	0.562
Peripheral artery disease	15	2.3	8	5.5	5	5.0	0.035	0.112	0.869
Congestive heart failure CHF (Previous)	23	3.5	20	13.7	10	10.0	<0.001	0.003	0.384
Smoking (current/previous)	414	62.6	79	54.1	57	57.0	0.065	0.272	0.697
Atrial fibrillation	95	14.8	42	29.4	26	26.0	<0.001	0.002	0.701
Hyperlipidemia	463	96.1	110	96.5	80	100	0.829	0.071	0.090
Hypertension	354	53.6	95	65.1	67	67.0	0.011	0.012	0.754
Diabetes	135	20.4	45	30.8	28	28.0	0.006	0.085	0.634

*p* values for comparison: i/e, included versus excluded; i/d, included versus declined; d/e, declined versus excluded. Hyperlipidemia: treatment initiated or untreated total cholesterol >4.5 mmol/L or untreated LDL cholesterol >2.5 mmol/L

*BMI* body mass index, *eGFR* estimated glomerular filtration rate, *IHD* ischemic heart disease, *LDL* low-density lipoprotein, *mRS* modified Rankin scale, *NSTEMI* non-ST-elevation myocardial infarction, *SD* standard deviation, *STEMI* ST-elevation myocardial infarction

Patients who declined participation had significantly different baseline characteristics than “included” patients. Declining patients were generally older with a larger proportion being women and patients with mRS >3, and a larger proportion lacked education above compulsory school level. Also, the eGFR was lower in the “declining” group. Regarding established risk factors, a larger proportion of patients in the group that declined participation had previous cerebrovascular disease, congestive heart failure CHF and atrial fibrillation. Patients who declined participation differed from excluded patients only in regards to a lower proportion with a mRS score >3 or education above compulsory school level, but these differences were not significant.

**Fig. 2:**
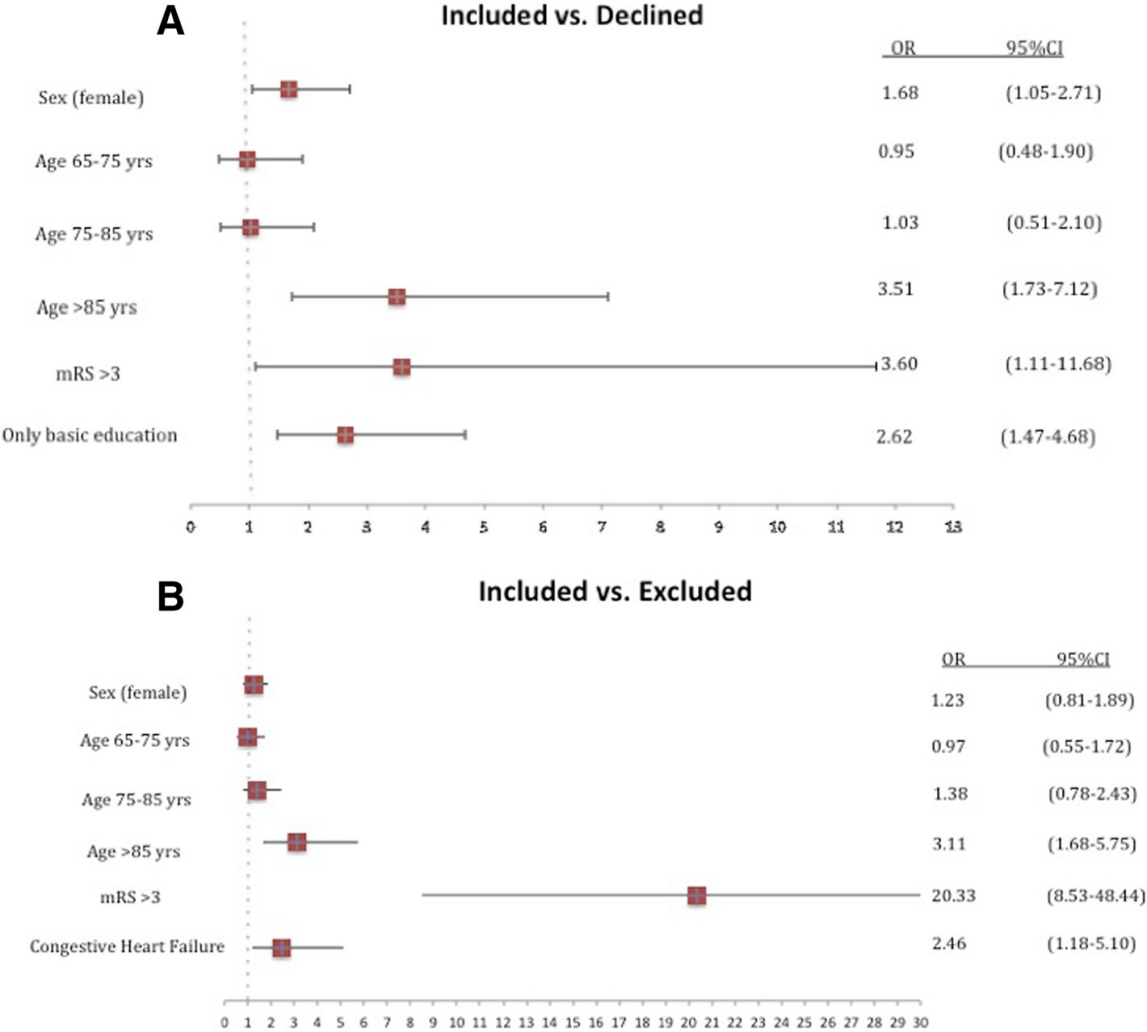
**a** Multivariate association with decision to decline participation. **b** With exclusion. Age 65 years or under was used as a reference category

**Fig. 3:**
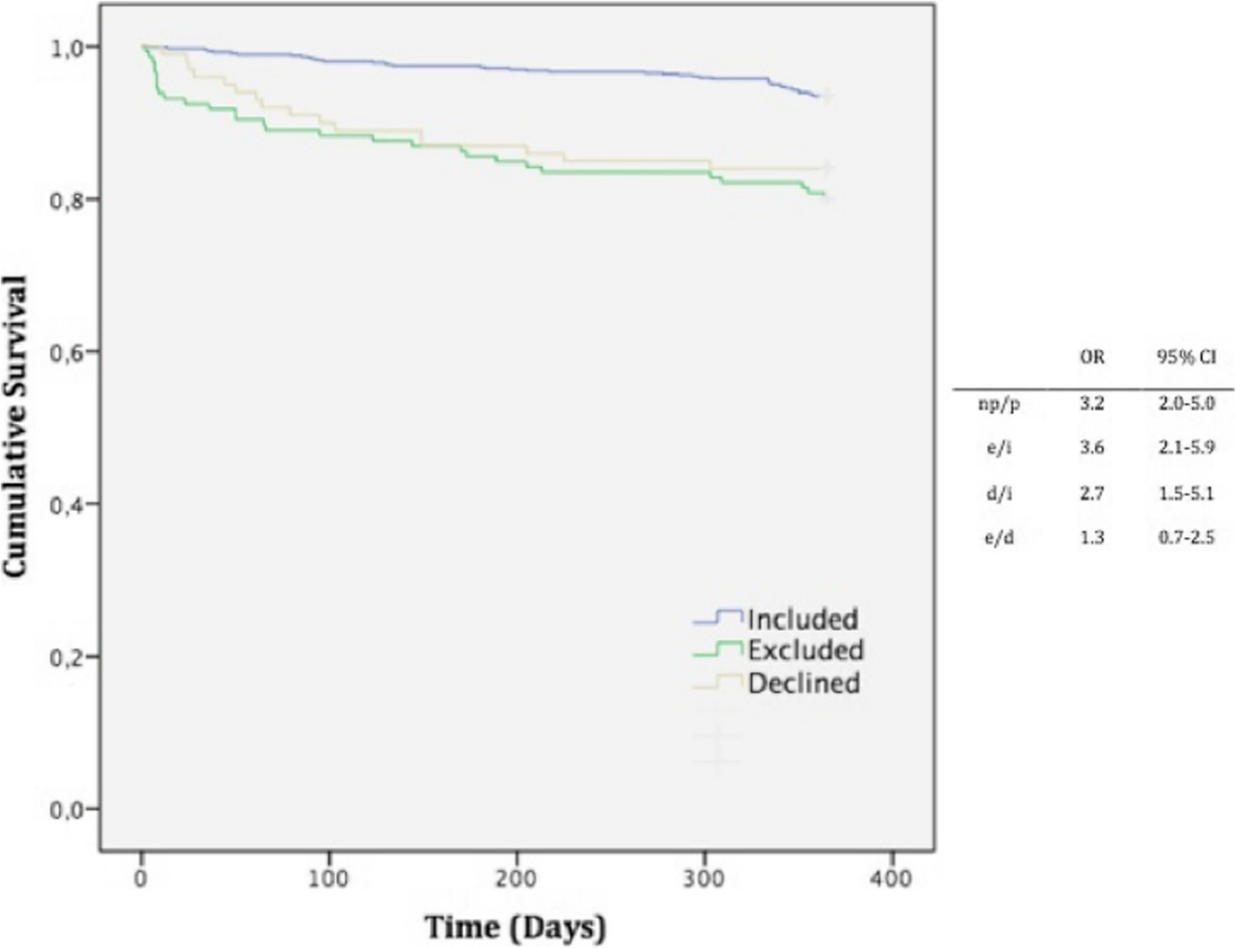
One-year survival Kaplan-Meier estimates and group comparison. Odds ratio (OR) for mortality. *p/np* participant/non-participant, *i* included, *e* excluded, *d* declined

In the first multivariate analysis, female sex, mRS >3, education limited to compulsory school level and age 85 years or older were significantly associated with a decision to decline participation (Fig. 2). In the second multivariate model, age 85 years or older, mRS >3 and known congestive heart failure CHF were associated with exclusion (Fig. 2). During the first year after discharge, 88 (9.7 %) patients died: 43 in the “included” group (6.5 %), 29 in the “excluded” group (19.9 %) and 16 in the “declining” group (16 %). Regarding the 1-year mortality prognosis, we found a significant difference between the “included” group and the “excluded” and “declining” groups (*p* <0.001, Fig. 3). The cumulative survival was not significantly different (*p* = 0.21) between the “excluded” and “declining” groups. However, the Kaplan-Meier curves revealed increased mortality during the first few months for the “excluded” group. Cardiovascular reasons were an insignificantly more common cause of death (52.1 % versus 47.9 %) than non-cardiovascular reasons in the overall population.

## Discussion

This study was a non-participation feasibility study of a telemedical method to improve adherence to secondary preventive measures after a coronary ischemic event. Almost three quarters of admitted patients were eligible for inclusion in our community-based rural cohort. The main reasons for non-participation were declining by one’s own will, participation in another medical trial, dementia and severe cardiovascular disease.

In univariate analyses, factors that indicated a larger proportion of co-morbidity (decreased eGFR and BMI and previous heart disease or CVL) were significant for non-participation and a disadvantage for women. We can only speculate about the result for women, but our subanalyses showed a significantly lower level of education among women compared to men (data not shown). Low education is considered a measure of socioeconomic status (SES) and known to increase cardiovascular risk [17].

Our study showed a significant increase in 1-year mortality for non-participants. Since the groups are selected as shown in Table 1, the mortality analysis is only descriptive and illustrates the high early mortality in non-participants. Subjective reasons for declining participation are multifaceted. We tried to objectify these reasons using a multivariate regression model of quantifiable surrogate variables. This analysis showed that the main variables for the decision to decline were older age, increased disability as measured in mRS, and education limited to compulsory school level. Predictors of exclusion in the second multivariate regression model were older age, reduced autonomy in mRS and congestive heart failure CHF. Notably, the “excluded” and “declined” groups only differed in terms of autonomy (mRS >3) at univariate comparison.

Reducing the burden of modifiable risk factors is an important goal for both individuals and society. Therefore, a standardized and cost-effective programme that can be applied to a large proportion of patients in both urban and rural settings is needed. We designed the NAILED study without predefined exclusion criteria except participation in another trial or inability to adhere to the concept of a telephone intervention. We intended to mimic a natural cohort and clinical setting as much as possible. We found that a simple follow-up method made it possible to include a large proportion of an unselected population-based ACS cohort.

To the best of our knowledge, this is the first study of the implementation of a multifactorial secondary preventive programme at the population level. Other studies on telephone-based secondary prevention programmes are restricted in terms of more extensive inclusion or exclusion criteria, age or small selected population samples [12, 18]. Consequently, direct comparisons regarding rates of participation and mortality are difficult.

A dilemma with existing secondary preventive programmes is that they tend to be more accessible to patients with higher income and education. The PURE study concluded that, even though high-income countries have the highest prevalence of cardiovascular risk factors, mortality is lower than in low-income countries due to less effective healthcare in the latter [19, 20]. In a study by Bergström et al., prognosis after AMI worsened with lower SES despite a state-funded healthcare system with a strong egalitarian tradition and after adjusting for traditional risk factors [21]. Jelinek et al. showed that individual coaching via telephone reduced inequalities in secondary preventive target fulfillment due to social class [22]. Eighteen months after the intervention, the effects were sustained [23].

The present study was conducted in a rural setting in Sweden. However, the present secondary prevention programme can be implemented in both urban and rural settings in large parts of the world as much of the intervention was by telephone with little demand for travel. Intervening health workers need only basic training and access to a consulting physician.

It is important that a secondary prevention strategy is designed so that all inhabitants can take part, regardless of age and co-morbidities. The NAILED protocol was constructed to be as including and comprehensive as possible. Because low education and reduced autonomy were independent factors for a patient’s unwillingness to participate, we speculate on the presence of an increased need for education at discharge in these groups. The older aged and advanced disease group is more challenging, but studies indicate low adverse effects from secondary preventive interventions and potential benefits on morbidity, but various effects on mortality [24, 25]. Further studies need to be conducted to elucidate with what means we can reach our oldest patients with multi-morbidity. It would also be important to identify settings in which preventive efforts are futile.

### Strengths and limitations

A strength of this study is that the cohort consists of an unselected cardiovascular population. Only one hospital is in the catchment area, which gives us a good overview of the cohort and local treatment traditions. During an initial 3-month control period, no missed cases were found. A weakness is that this is a single center study and general applicability may be questioned. We also lack variables to extensively evaluate SES. Additionally, patients not referred to the cardiology department, probably due to clinicians regarding interventions as more harmful than useful in the specific patient’s circumstances, could have been missed despite the study nurses surveying all departments outside the cardiac care unit.

## Conclusion

Nurse-led telephone-based follow-up of secondary prevention after coronary ischemic events can be applied to a large proportion of patients in an unselected clinical setting. Increased mortality is seen in those who do not participate. Reasons for non-participation include older age, multiple co-morbidities, decreased functional status and education limited to compulsory school level.

## Abbreviations

ACS: acute coronary syndrome
BMI: body mass index
CHF: congestive heart failure
CI: confidence interval
CVL: cerebrovascular lesion
eGFR: estimated glomerular filtration rate
GP: general practitioner
IHD: ischemic heart disease
mRS: modified Rankin scale
NAILED: Nurse-based Age-independent Intervention to Limit Evolution of Disease
NSTEMI: non-ST-elevation myocardial infarction
OR: odds ratio
SES: socioeconomic status
STEMI: ST-elevation myocardial infarction
UA: unstable angina

## References

[1] Smith SC, Collins A, Ferrari R, Holmes DR, Logstrup S, McGhie DV (2012). Our time: a call to save preventable death from cardiovascular disease (heart disease and stroke). Circulation.

[2] Rossini R, Capodanno D, Lettieri C, Musumeci G, Limbruno U, Molfese M (2013). Long-term outcomes of patients with acute coronary syndrome and nonobstructive coronary artery disease. Am J Cardiol.

[3] Bjorck L, Rosengren A, Bennett K, Lappas G, Capewell S (2009). Modelling the decreasing coronary heart disease mortality in Sweden between 1986 and 2002. Eur Heart J.

[4] Jernberg T, Johanson P, Held C, Svennblad B, Lindback J, Wallentin L (2011). Association between adoption of evidence-based treatment and survival for patients with ST-elevation myocardial infarction. JAMA.

[5] Perk J, De Backer G, Gohlke H, Graham I, Reiner Z, Verschuren M (2012). European Guidelines on cardiovascular disease prevention in clinical practice (version 2012). The Fifth Joint Task Force of the European Society of Cardiology and Other Societies on Cardiovascular Disease Prevention in Clinical Practice (constituted by representatives of nine societies and by invited experts). Eur Heart J.

[6] Rockson SG, deGoma EM, Fonarow GC (2007). Reinforcing a continuum of care: in-hospital initiation of long-term secondary prevention following acute coronary syndromes. Cardiovasc Drugs Ther.

[7] Bhatt DL, Steg PG, Ohman EM, Hirsch AT, Ikeda Y, Mas JL (2006). International prevalence, recognition, and treatment of cardiovascular risk factors in outpatients with atherothrombosis. JAMA.

[8] Cacoub PP, Zeymer U, Limbourg T, Baumgartner I, Poldermans D, Rother J (2011). Effects of adherence to guidelines for the control of major cardiovascular risk factors on outcomes in the REduction of Atherothrombosis for Continued Health (REACH) Registry Europe. Heart.

[9] SWEDEHEART. Annual report 2014. http://www.ucr.uu.se/swedeheart/index.php/dokument-sh/arsrapporter. Accessed 22 January 2015.

[10] Kotseva K, Wood D, De Bacquer D, De Backer G, Ryden L, Jennings C (2015). EUROASPIRE IV: a European Society of Cardiology survey on the lifestyle, risk factor and therapeutic management of coronary patients from 24 European countries. Eur J Prev Cardiol.

[11] Gielen S, Landmesser U (2014). The Year in Cardiology 2013: cardiovascular disease prevention. Eur Heart J.

[12] Neubeck L, Redfern J, Fernandez R, Briffa T, Bauman A, Freedman SB (2009). Telehealth interventions for the secondary prevention of coronary heart disease: a systematic review. Eur J Cardiovasc Prev Rehabil.

[13] Kotb A, Hsieh S, Wells GA (2014). The effect of telephone support interventions on coronary artery disease (CAD) patient outcomes during cardiac rehabilitation: a systematic review and meta-analysis. PLoS One.

[14] Bernocchi P, Scalvini S, Bertacchini F, Rivadossi F, Muiesan ML (2014). Home based telemedicine intervention for patients with uncontrolled hypertension – -a real life non-randomized study. BMC Med Inform Decis Mak..

[15] Mooe T, Bjorklund F, Graipe A, Huber D, Jakobsson S, Kajermo U (2014). The Nurse-Based Age Independent Intervention to Limit Evolution of Disease After Acute Coronary Syndrome (NAILED ACS) Risk Factor Trial: protocol for a randomized controlled trial. JMIR Res Protoc.

[16] Thygesen K, Alpert JS, White HD (2007). Joint ESCAAHAWHFTFftRoMI. Universal definition of myocardial infarction. J Am Coll Cardiol.

[17] Winkleby MA, Jatulis DE, Frank E, Fortmann SP (1992). Socioeconomic status and health: how education, income, and occupation contribute to risk factors for cardiovascular disease. Am J Public Health.

[18] Clark AM, Haykowsky M, Kryworuchko J, MacClure T, Scott J, DesMeules M (2010). A meta-analysis of randomized control trials of home-based secondary prevention programs for coronary artery disease. Eur J Cardiovasc Prev Rehabil.

[19] Yusuf S, Islam S, Chow CK, Rangarajan S, Dagenais G, Diaz R (2011). Use of secondary prevention drugs for cardiovascular disease in the community in high-income, middle-income, and low-income countries (the PURE Study): a prospective epidemiological survey. Lancet.

[20] Chow CK, Teo KK, Rangarajan S, Islam S, Gupta R, Avezum A (2013). Prevalence, awareness, treatment, and control of hypertension in rural and urban communities in high-, middle-, and low-income countries. JAMA.

[21] Bergstrom G, Redfors B, Angeras O, Dworeck C, Shao Y, Haraldsson I (2014). Low socioeconomic status of a patient’s residential area is associated with worse prognosis after acute myocardial infarction in Sweden. Int J Cardiol..

[22] Jelinek MV, Santamaria JD, Best JD, Thompson DR, Tonkin AM, Vale MJ (2014). Reversing social disadvantage in secondary prevention of coronary heart disease. Int J Cardiol.

[23] Jelinek M, Vale MJ, Liew D, Grigg L, Dart A, Hare DL (2009). The COACH program produces sustained improvements in cardiovascular risk factors and adherence to recommended medications – two years follow-up. Heart Lung Circ.

[24] Bejan-Angoulvant T, Saadatian-Elahi M, Wright JM, Schron EB, Lindholm LH, Fagard R (2010). Treatment of hypertension in patients 80 years and older: the lower the better? A meta-analysis of randomized controlled trials. J Hypertens.

[25] Strandberg TE, Kolehmainen L, Vuorio A (2014). Evaluation and treatment of older patients with hypercholesterolemia: a clinical review. JAMA.

